# Understanding the evolution of epistemic emotions research: a bibliometric review from 2005 to 2024

**DOI:** 10.3389/frma.2026.1852187

**Published:** 2026-08-28

**Authors:** Jaya Shukla, Ram Manohar Singh

**Affiliations:** 1 Jindal Institute of Behavioural Sciences, O. P. Jindal Global University, Sonipat, India; 2 Department of Humanities and Social Sciences, Indian Institute of Technology Roorkee, Roorkee, India

**Keywords:** bibliometric analysis, control-value theory, educational psychology, epistemic emotions, students

## Abstract

**Introduction:**

Epistemic emotions play a central role in fostering cognitive engagement and knowledge construction. Although research on epistemic emotions has expanded considerably over the past two decades, a comprehensive synthesis of the field's intellectual structure, thematic development, and emerging researchdirections has been lacking.

**Methods:**

This study addresses this gap through a bibliometric analysis of 185 publications indexed in Scopus and Web of Science between 2005 and 2024 using Biblioshiny in R. The analysis examined publication trends, influential authors, journals, institutions, countries, collaboration networks, and thematic evolution to provide a comprehensive overview of the field.

**Results:**

The findings reveal sustained growth in scholarly output, particularly after 2016, reflecting the increasing recognition of epistemic emotions within educational psychology, cognitive science, and related disciplines. Thematic analysis indicates a strong theoretical foundation centered on achievement emotions, Control-Value Theory, and knowledge construction, alongside growing methodological innovation through affective computing and emotion recognition technologies.

**Discussion:**

This review provides a comprehensive synthesis of the intellectual development of epistemic emotions research and identifies strategic priorities for future research, including greater theoretical integration, methodological innovation, cross-cultural investigations, and technology-enhanced learning applications.

## 1 Introduction

Epistemic emotions, a subset of academic emotions centered around the pursuit, evaluation, and integration of knowledge, play a significant role in learning and cognitive processes (Pekrun and Linnenbrink-Garcia, 2014). These emotions, such as curiosity, surprise, confusion, etc. are essential for guiding attention, promoting deep engagement, and enhancing cognitive performance (Pekrun and Stephens, 2012; Muis et al., 2018a,b). Researchers have operationalized epistemic emotions as an affective state related to knowledge acquisition and generation (Pekrun and Stephens, 2012), emerging from an information-oriented appraisal concerning the alignment or misalignment between new information and existing beliefs (Vogl et al., 2019; McPhetres, 2019; Muis et al., 2018a,b; Pekrun and Linnenbrink-Garcia, 2014). They are tied to the knowledge-producing aspects of cognitive tasks and activities (Morton, 2010) and are pivotal in learning, conceptual change, and cognitive performance (Pekrun and Stephens, 2012; Pekrun et al., 2017a,b), influencing cognitive and metacognitive strategies (Tornare et al., 2015; Trevors et al., 2017). They typically arise when the learning objective is knowledge-seeking (Muis et al., 2018a,b), such as during the execution of complex cognitive tasks, learning a new technique, understanding a situation, solving a problem, or more broadly, acquiring knowledge about a topic (Nerantzaki et al., 2021). According to epistemic emotion theories, they arise when individuals encounter novel, complex, or ambiguous information, prompting them to engage with the material more deeply (Chevrier et al., 2019).

In educational contexts, epistemic emotions are seen as both precursors and outcomes of effective learning strategies (Pekrun et al., 2017a,b). Studies have shown that emotions like curiosity foster exploration, while emotions like confusion can signal cognitive dissonance, encouraging learners to resolve discrepancies in their understanding (D'Mello and Graesser, 2012). Given the impact of these emotions on academic performance, educational researchers are increasingly examining the mechanisms by which epistemic emotions influence knowledge acquisition and conceptual change (Vogl et al., 2019). This emphasis aligns with broader shifts in education and psychology that seek to understand the interconnected roles of cognitive, motivational, and emotional factors in learning (Pekrun et al., 2017a,b).

Over the past two decades, the field of epistemic emotions has witnessed substantial growth. Scholars have progressively moved from conceptual frameworks (e.g., Pekrun et al., 2006; Morton, 2010) to empirical studies that link epistemic emotions with various cognitive and metacognitive outcomes (Trevors et al., 2017; Vogl et al., 2019). Early studies focused on defining and categorizing epistemic emotions, proposing theoretical models that conceptualized these emotions as responses to cognitive and knowledge-related challenges (Pekrun et al., 2006). Subsequent research has expanded to examine epistemic emotions across diverse educational settings, from classrooms to digital learning environments (Arguel et al., 2019). A considerable body of literature has investigated the role of epistemic emotions in promoting engagement with complex tasks, enhancing motivation, and supporting self-regulated learning (Pekrun et al., 2017a,b). The development of Epistemically-Related Emotion Scales (EES) by Pekrun et al. (2017a,b) is a notable milestone, providing a tool for measuring epistemic emotions in educational research. The EES has facilitated a more nuanced understanding of how emotions like curiosity, confusion, and surprise relate to learning outcomes and academic success (Pekrun et al., 2017a,b). Studies have also shown that epistemic emotions significantly affect learners' problem-solving strategies, decision-making processes, and persistence in challenging tasks (D'Mello et al., 2014; Vilhunen et al., 2022).

While epistemic emotions research is rooted in educational psychology, the concept has attracted attention from various fields, including cognitive science, neuroscience, and educational technology (Vogl et al., 2019; Chevrier et al., 2019; Dubey and Griffiths, 2020). In cognitive science, researchers are investigating the neural mechanisms underlying epistemic emotions, exploring how brain regions associated with motivation and reward are activated in response to curiosity-driven learning (Murayama, 2022). Neuroscientific studies have contributed to a deeper understanding of the physiological basis of epistemic emotions, highlighting the role of dopaminergic pathways in curiosity and motivation (Jepma et al., 2012). Educational technology researchers, on the other hand, are examining how digital tools can evoke epistemic emotions to enhance online learning. Interactive environments and adaptive learning systems are designed to introduce elements of surprise and challenge, thereby stimulating curiosity and engagement (Arguel et al., 2019). These interdisciplinary applications illustrate the widespread relevance of epistemic emotions and their potential to improve educational practices.

Although empirical studies on epistemic emotions have increased rapidly, the field lacks a comprehensive bibliometric synthesis that maps its intellectual structure, collaborative networks, and thematic evolution. Addressing this gap, bibliometric analysis provides a structured approach to map the intellectual structure and evolution of research in epistemic emotions (Aria and Cuccurullo, 2017). Through bibliometric analysis, prominent authors, influential journals, high-impact articles, and collaborative networks that shape epistemic emotions research are identified (Thangavel and Chandra, 2023). A bibliometric review of epistemic emotions is especially relevant at this stage, given the rapid growth of the field over recent years (Pekrun, 2022). By analyzing a dataset of publications from 2005 to 2024, we aim to provide a comprehensive analysis of the trends and trajectory of epistemic emotions.

The main objectives of this bibliometric review are threefold. First, this study seeks to identify the most productive journals, authors, and institutions in epistemic emotions research, providing a quantitative overview of the field's key contributors. Second, the review examines publication trends, analyzing the growth rate and citation impact of research on epistemic emotions. This analysis helps to pinpoint influential scholars that have shaped the field and to recognize recent publications that are advancing new research directions (Aria and Cuccurullo, 2017). Finally, the study explores thematic trends within epistemic emotions research, using keyword analysis to identify emerging themes that reflect current research priorities. Specifically, this study seeks to address the following questions:

RQ1: What are the publication trends in epistemic emotions research from 2005 to 2024?
RQ2: Who are the leading authors, institutions, and countries in the field?
RQ3: Which journals and publications have significantly influenced the field's development?
RQ4: How do collaborative networks and geographical contributions shape epistemic emotions research?
RQ5: What are the core themes and emerging trends in epistemic emotions research?

Through these questions, the paper identifies the intellectual landscape of epistemic emotions research, highlighting its growth trajectory, influential contributors, and emerging thematic directions. Given the importance of emotions in learning processes, understanding the academic landscape of epistemic emotions research is crucial for advancing educational practices that promote cognitive and emotional engagement. Also, this study offers a practical framework for scholars interested in conducting bibliometric analysis, demonstrating the capabilities of the Biblioshiny tool in identifying research trends and thematic areas. The findings aim to provide researchers with a comprehensive overview of the field, guiding future investigations into the role of epistemic emotions in educational contexts and beyond.

## 2 Methodology

Bibliometric analysis is a well-established quantitative approach for evaluating the intellectual structure, research trends, and scientific development of a research field (Donthu et al., 2021; Pritchard and Wittig, 1981). This analytical approach has become increasingly popular for studying the development of academic disciplines, offering a structured way to map scientific trends. In the context of educational psychology, bibliometric reviews have been utilized to explore diverse areas such as emotional intelligence, self-concept, motivation, personality and achievement among students (Sharma and Tiwari, 2024). However, the exploration of epistemic emotions through bibliometric analysis remains underdeveloped, highlighting the need for a comprehensive review.

For this review, bibliometric data were sourced from two major scientific databases, Scopus and Web of Science (WOS), following the PRISMA guidelines. These databases were selected because of their extensive coverage of high-quality peer-reviewed literature, and their widespread use in bibliometric research, thereby enhancing the reliability and comprehensiveness of science mapping analyses (Donthu et al., 2021; Aria and Cuccurullo, 2017).

**Figure 1:**
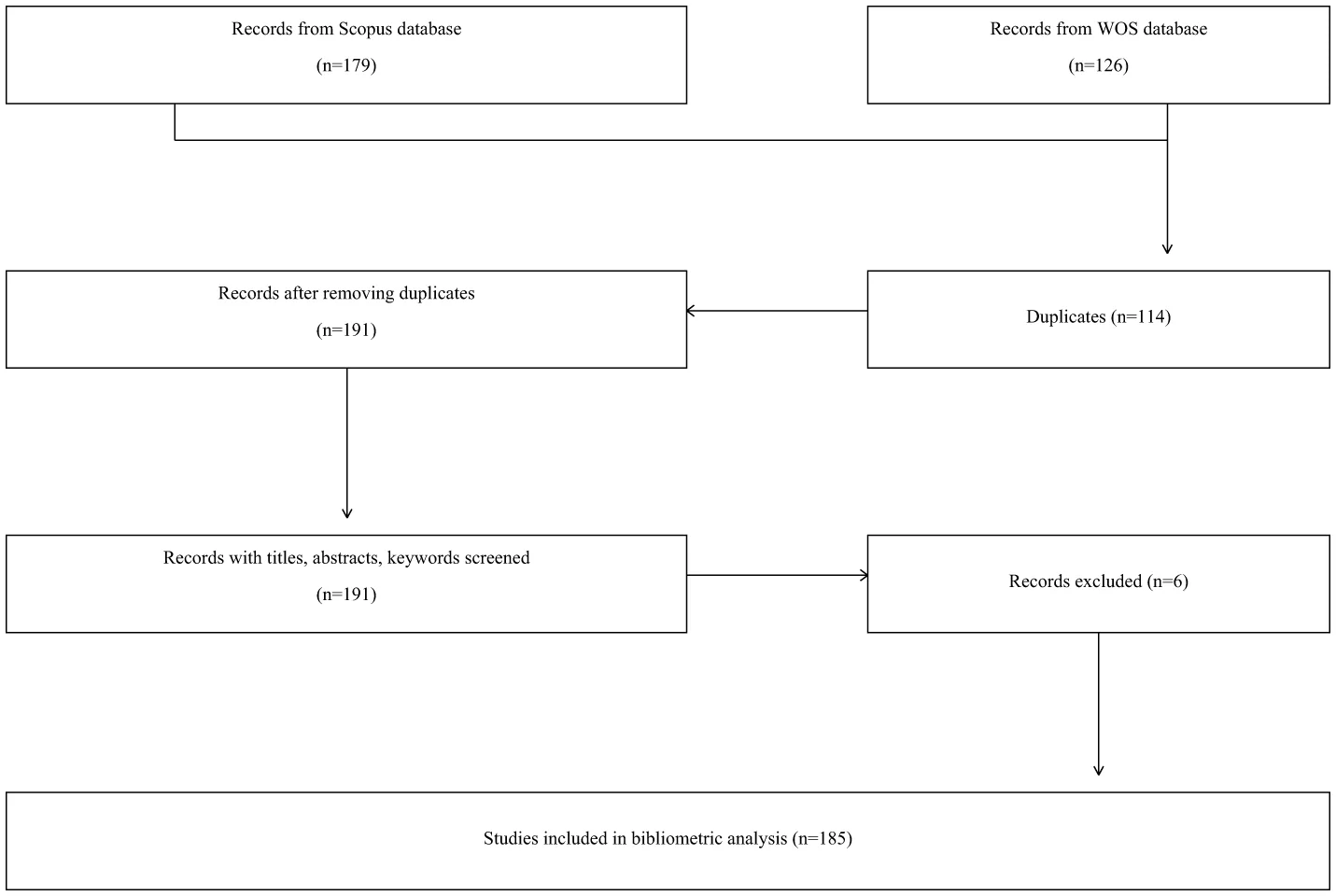
Framework of article selection process for bibliometric analysis as per PRISMA.

The search query was conducted on September 22, 2024, using the following search terms: “Epistemic Emotion,” OR “Epistemic Emotions,” OR “Epistemic Emotion^*^” within the titles, abstracts, and keywords of publications. The framework of article selection process is presented in Figure 1. The scope was restricted to documents published in English to ensure consistency in analysis. The initial search yielded a total of 305 records, with 179 documents from Scopus and 126 from WOS. These records were subsequently merged, and duplicate entries were identified and removed, resulting in a dataset of 191 unique publications. Further screening involved an examination of titles, abstracts, and keywords, leading to the exclusion of six additional documents that did not align with the focus of the review. Ultimately, 185 publications were retained for in-depth bibliometric analysis.

The final dataset was analyzed using Biblioshiny, an advanced open-source tool designed for comprehensive science mapping, integrated within the R environment (Aria and Cuccurullo, 2017). Biblioshiny was selected because it provides a comprehensive and reproducible framework for performance analysis and science mapping, integrating descriptive, network, and thematic analyses within a single platform (Donthu et al., 2021). This selection was driven by its robust capabilities in visualizing bibliometric networks and generating detailed reports.

## 3 Results

### 3.1 General information of the publication collection

The main information of the 185 documents published between 2005 and 2024 is presented in Table 1. Among the document types, articles constitute the majority of the contributions, accounting for 151 documents (81.62%). This dominance highlights the centrality of research articles as the primary medium for disseminating findings in this field. Book chapters (*n* = 12) conference papers (*n* = 11), review papers (*n* = 9), and meeting abstract (*n* = 2) together accounted for 18.38% of total publications. Though the presence of review papers is limited, it intends to provide valuable synthesis and reflection on existing knowledge, while conference papers, meeting abstracts and book chapters indicate emerging themes and ongoing debates in the field. These 185 documents have been published in 141 different sources, including journals, books, and conference proceedings. The publication collection has been written by 455 different authors (on average each document has three co-authors). There were 36 single authors who published 42 single-authored documents. The average citations per document is 15.69, demonstrating moderate citation activity. The average age of the documents is 2.63 years, signifying that many of the papers in this dataset are relatively recent. This implies that research on epistemic emotions is still evolving, with a significant portion of the literature being published within the last few years. The 25.41% of international co-authorship in the dataset highlights the widespread academic interest in epistemic emotions across countries and institutions, fostering diverse perspectives and enhancing the global discourse on the topic.

**Table 1:** Main information of the publication collection.

Description	Result
Articles	151 (81.62%)
Book chapters	12 (6.5%)
Conference papers	11 (5.94%)
Review papers	9 (4.86%)
Meeting abstract	2 (1.08%)
Sources (journals, books, etc.)	141
Period	2005–2024
Average citations per document	15.69
Authors	455
Authors of single-authored document	36
Single-authored documents	42
Co-authors per document	3.23
International co-authorships %	25.41
Total documents	185
Annual growth rate %	20.58
Document average age	2.63
Average citations per doc	15.69

**Figure 2:**
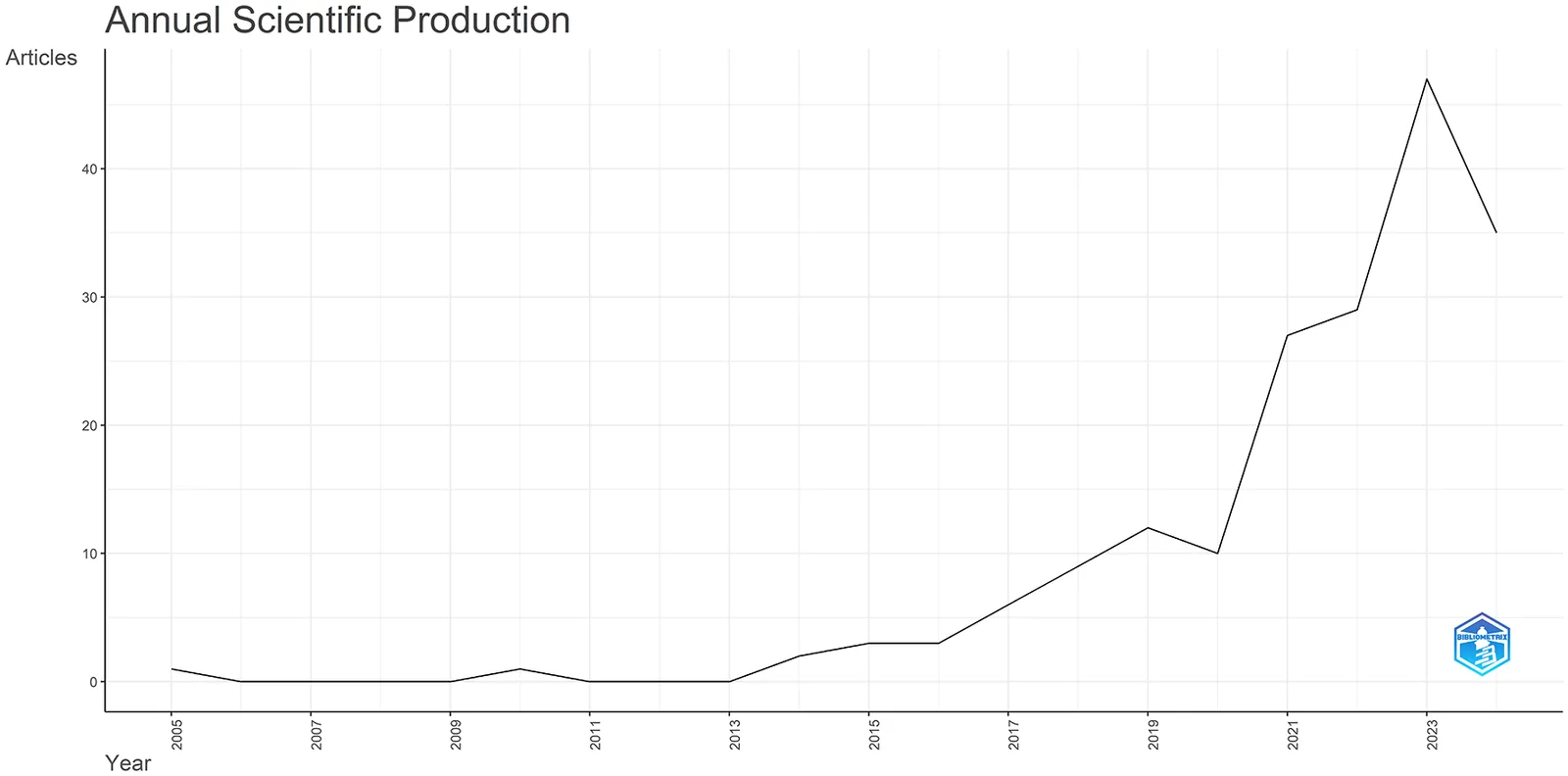
The publication trend of the publication collection (2005–2024).

The publication trend, as depicted in Figure 2, shows that the earliest study on epistemic emotions in the dataset appeared in 2005. Research activity remained limited through 2016, followed by a marked upward shift beginning in 2017. The field experienced a substantial surge in 2023, reaching over 45 published works. By September 2024, 35 articles had already been published, indicating continued growth. This upward trajectory highlights the rapid expansion of epistemic emotions research in recent years. The trend suggests that research on epistemic emotions is becoming more prominent, likely due to its increasing relevance in areas like education, psychology, and cognitive sciences. This growth is aligned with the 20.58% annual growth rate of the field, signifying its evolving and expanding body of knowledge.

### 3.2 Most productive journals and their impact

The collected publications were distributed across 141 different sources, and the top ten journals that published the most papers related to epistemic emotions are presented in Table 2. The journal Frontiers in Education, with five publications, is ranked first, followed by Frontiers in Psychology, Journal of Educational Psychology, and Learning and Instruction, with four publications each. Other key journals include Cognition and Emotion, Computers in Human Behavior, Contemporary Educational Psychology, Discourse Processes, Emotion, and the International Journal of Science Education, each contributing three publications. The majority of these sources are classified under Q1 in the Scopus quartile and indexed in the Social Science Citation Index (SSCI). Publishers such as Frontiers, Elsevier, APA, and T&F have the highest presence in the top 10 sources.

**Table 2:** Top 10 most productive journals based on the number of publications.

Rank	Sources	No. of articles	Publisher/country	Scopus quartile	WOS core collection
1	FRONTIERS IN EDUCATION	5	Frontiers/Switzerland	Q2	ESCI
2	FRONTIERS IN PSYCHOLOGY	4	Frontiers/Switzerland	Q1	SSCI
3	JOURNAL OF EDUCATIONAL PSYCHOLOGY	4	APA/USA	Q1	SSCI
4	LEARNING AND INSTRUCTION	4	Elsevier/UK	Q1	SSCI
5	COGNITION & EMOTION	3	T&F/UK	Q1	SSCI
6	COMPUTERS IN HUMAN BEHAVIOR	3	Elsevier/Netherlands	Q1	SSCI
7	CONTEMPORARY EDUCATIONAL PSYCHOLOGY	3	Elsevier/USA	Q1	SSCI
8	DISCOURSE PROCESSES	3	T&F/UK	Q1	SSCI
9	EMOTION	3	APA/USA	Q1	SSCI
10	INTERNATIONAL JOURNAL OF SCIENCE EDUCATION	3	T&F/UK	Q1	SSCI

**Figure 3:**
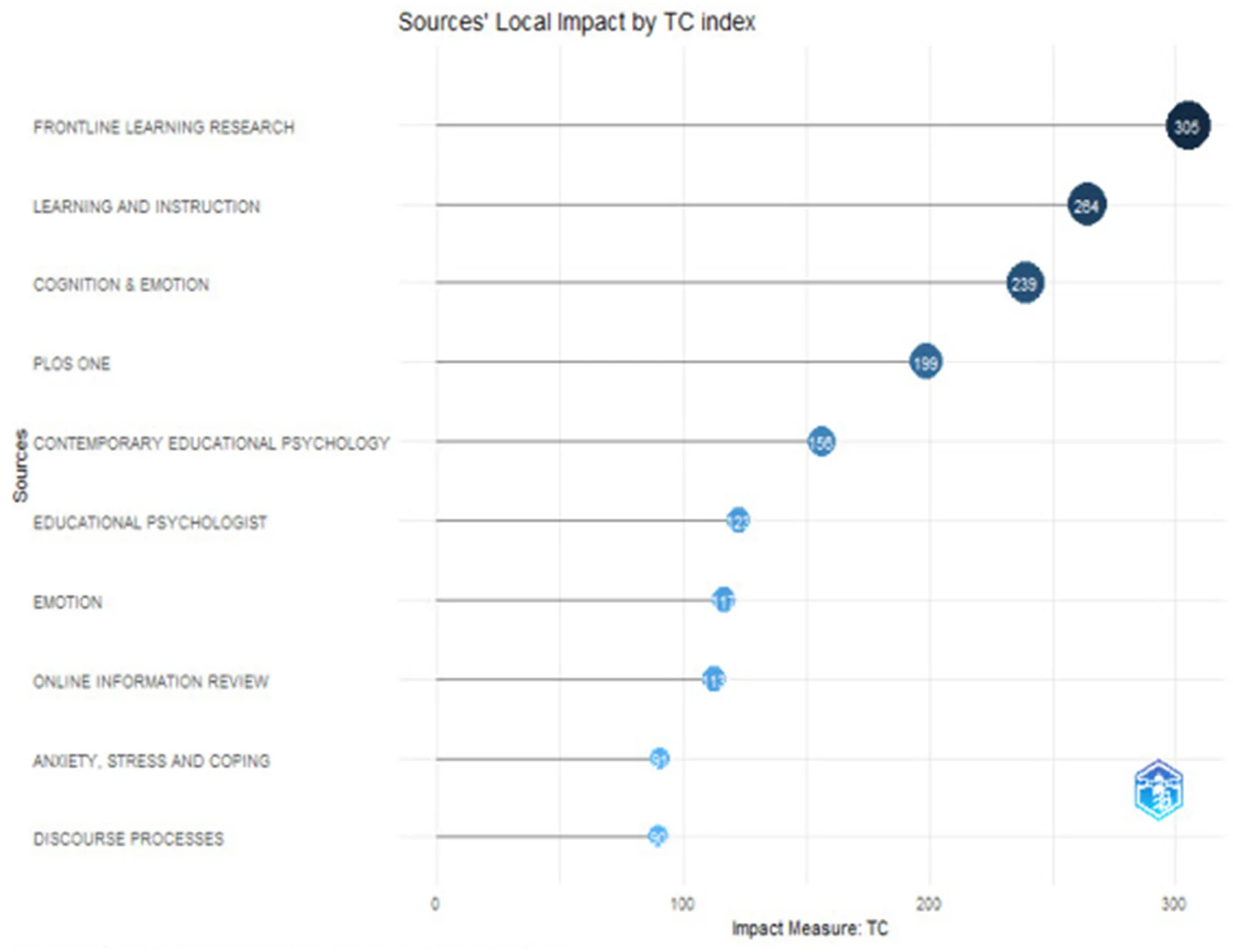
Top 10 sources' local impact by total citations.

Figure 3 shows the top ten sources' local impact ranked by total citations in the field of epistemic emotions. Frontline Learning Research ranks first with the highest number of citations (305), followed by Learning and Instruction (264 citations) and Cognition and Emotion (239 citations). PLOS ONE is ranked fourth with 199 citations, while Contemporary Educational Psychology and Educational Psychologist follow with 156 and 123 citations respectively. Other journals among top 10 list include Emotion (117 citations), Online Information Review (113 citations), Anxiety, Stress, and Coping (91 citations), and Discourse Processes (90 citations).

### 3.3 Prominent authors

Top 10 most productive authors in the field of epistemic emotions are presented in Table 3. The result reveals a diverse group of scholars from prominent institutions across the UK, USA, Canada, and Germany. Reinhard Pekrun, from the University of Essex, UK, tops the list with 17 publications, 1,049 citations, and an H-index of 13 indicating his significant impact and consistent contributions since his first publication in 2014. Krista Muis from McGill University, Canada, follows closely with 14 publications and 888 citations, and Gale Sinatra from the University of Southern California, USA, with 10 publications and 616 citations. Authors such as Marianne Chevrier and Gregory Trevors have contributed six publications each, while more recent scholars such as, Murayama et al. (2019) and Thacker et al. (2020) are steadily building their presence with five publications each. Other authors include Elisabeth Vogl, Kristina Loderer, and Jari Lavonen, who have contributed four publications each, with Jari Lavonen being the newest addition to the group, having started in 2021.

**Table 3:** Top 10 prominent authors based on the number of publications.

Authors	Institution/country	No. of publications	Total citations	H-index	Year of first publication
Reinhard Pekrun	University of Essex/UK	17	1,049	13	2014
Krista Muis	McGill University/Canada	14	888	11	2015
Gale Sinatra	University of Southern California/USA	10	616	8	2015
Marianne Chevrier	McGill University/Canada	6	389	6	2015
Gregory Trevors	University of South Carolina/USA	6	356	4	2015
Kou Murayama	University of Tübingen/Germany	5	218	4	2019
Ian Thacker	University of Texas/USA	5	86	3	2020
Elisabeth Vogl	Ludwig Maximilian University of Munich/Germany	4	388	4	2017
Kristina Loderer	University of Munich/Germany	4	225	4	2019
Jari Lavonen	University of Helsinki/Finland	4	42	4	2021

**Figure 4:**
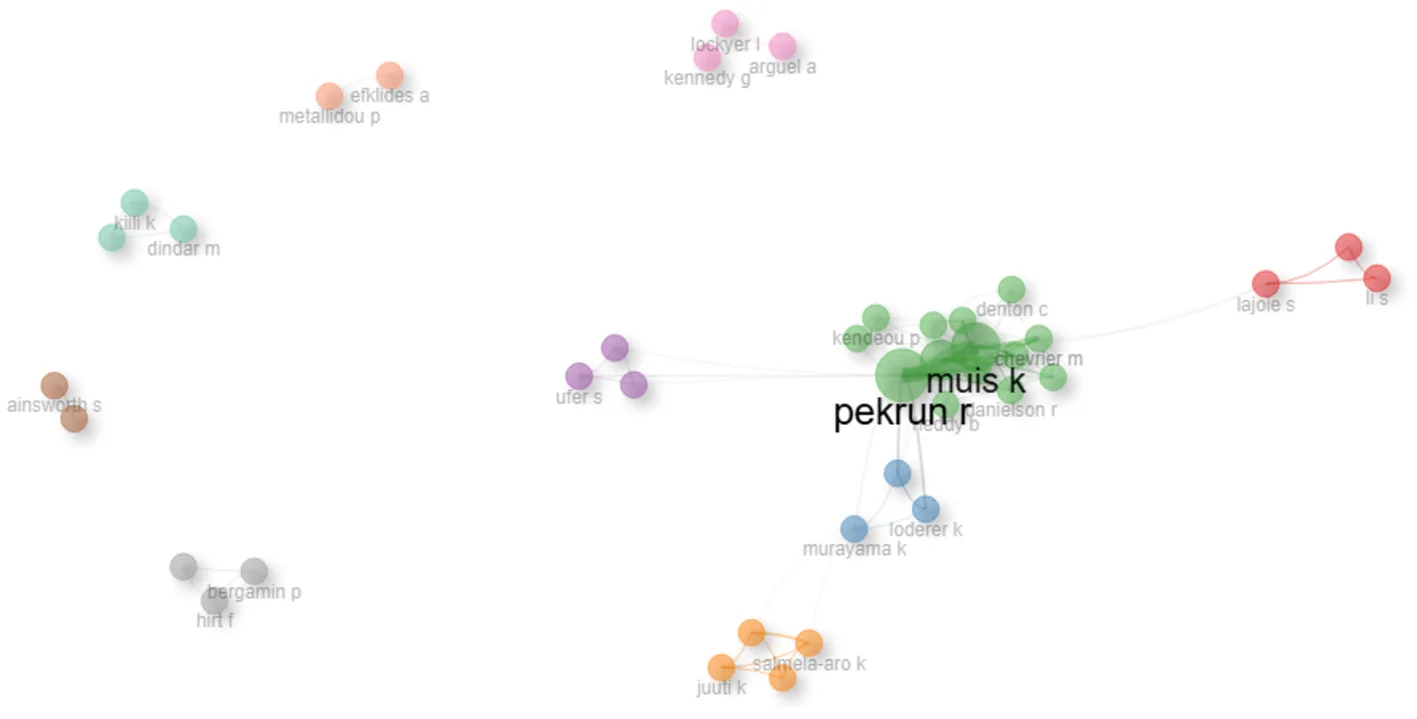
Collaboration network of authors.

Figure 4 illustrates the interconnected relationships among prominent researchers in the field of epistemic emotions. In this network, each node denotes an author, with the node size reflecting the relative publication count, while edges represent co-authorships between authors. The color-coded clusters identify distinct groups of researchers who frequently collaborate, pointing to sub-communities within the field. A notable cluster, represented in green, centers around Pekrun R, indicating their substantial collaborative network and influence in epistemic emotions research. Other clusters, such as those in red, orange, and purple, represent smaller but cohesive research teams focusing on specific subdomains. The network also shows isolated clusters with minimal connections to other groups, which may suggest specialized research areas or emerging subfields. Overall, this figure highlights the collaborative nature of authors studying epistemic emotions and highlights the potential for further cross-group collaborations to enhance the integration of knowledge across the field.

### 3.4 Top global cited articles and their theme

The Table 4 highlights top 20 research articles in epistemic emotions, with Schindler et al. (2017) leading at 199 citations for their work on measuring aesthetic emotions in PLOS ONE journal. Fischer et al. (2014) and Muis et al. (2015a,b) follow with 191 and 190 citations respectively. Other notable contributions include Pekrun et al. (2017a,b), Muis et al. (2018b) and Vogl et al. (2020). These most cited articles in epistemic emotions research reflect several key themes central to understanding their role in learning and cognition. A primary focus is on the measurement of epistemic emotions, with works by Schindler et al. (2017) and Pekrun et al. (2017a,b) developing tools to assess emotions like curiosity and confusion in learning contexts. Another significant theme is the influence of epistemic emotions on learning and reasoning, as explored by Fischer et al. (2014) and Muis et al. (2015a,b), who highlight how these emotions shape strategies, scientific reasoning, and argumentation. Studies by Muis et al. (2018a) and Zheng et al. (2021) investigate the interplay between emotions and self-regulation, examining how emotional states impact personal motivation and learning outcomes. Research by Arguel et al. (2019) and Chevrier et al. (2019) explores the management of epistemic emotions within digital and interactive learning environments, highlighting their relevance in modern educational technology. Finally, the role of epistemic emotions in facilitating conceptual change is addressed in studies by Trevors et al. (2016) and Thacker et al. (2020), which explore how emotions contribute to belief revision and adaptive learning. These themes highlight the importance of epistemic emotions in shaping educational experiences and advancing cognitive development.

**Table 4:** Top 20 global cited articles.

Paper	Paper title	DOI	Total citations
Schindler et al. (2017), PLOS ONE	Measuring aesthetic emotions: a review of the literature and a new assessment tool	10.1371/journal.pone.0178899	199
Fischer et al. (2014), FRONTLINE LEARN RES	Scientific Reasoning and Argumentation: advancing an Interdisciplinary Research Agenda in Education	10.14786/flr.v2i3.96	191
Muis et al. (2015a), LEARN INSTR	The curious case of climate change: testing a theoretical model of epistemic beliefs, epistemic emotions, and complex learning	10.1016/j.learninstruc.2015.06.003	190
Pekrun et al. (2017b), COGN EMOT	Measuring emotions during epistemic activities: the Epistemically-Related Emotion Scales	10.1080/02699931.2016.1204989	179
Muis et al. (2018a), EDUC PSYCHOL	The role of epistemic emotions in personal epistemology and self-regulated learning	10.1080/00461520.2017.1421465	123
Muis et al. (2015b) CONTEMP EDUC PSYCHOL	The role of epistemic emotions in mathematics problem solving	10.1016/j.cedpsych.2015.06.003	120
Vogl et al. (2020), EMOTION	Surprised–curious–confused: epistemic emotions and knowledge exploration	10.1037/emo0000578	115
Heitzmann et al. (2019), FRONTLINE LEARN RES	Facilitating diagnostic competences in simulations: a conceptual framework and a research agenda for medical and teacher education	10.14786/flr.v7i4.384	114
Miceli and Castelfranchi (2005), ANXIETY STRESS COPING	Anxiety as an “epistemic” emotion: an uncertainty theory of anxiety	10.1080/10615800500209324	91
Morton (2010), THE OXF HANDB OF PHILOS OF EMOT	Epistemic emotions	10.1093/oxfordhb/9780199235018.003.0018	89
Arguel et al. (2019), INTERACT LEARN ENVIRON	Seeking optimal confusion: a review on epistemic emotion management in interactive digital learning environments	10.1080/10494820.2018.1457544	87
Trevors et al. (2016), DISCL PROCESS	Identity and epistemic emotions during knowledge revision: a potential account for the backfire effect	10.1080/0163853X.2015.1136507	74
Zheng et al. (2021), COMPUT EDUC	Self-regulation and emotion matter: a case study of instructor interactions with a learning analytics dashboard	10.1016/j.compedu.2020.104061	71
Vogl et al. (2019), FRONT PSYCHOL	Surprise, curiosity, and confusion promote knowledge exploration: evidence for robust effects of epistemic emotions	10.3389/fpsyg.2019.02474	69
Pekrun (2016), METHODOLOGICAL ADVANCES IN RESEARCH ON EMOTION AND EDUCATION, Springer	Using self-report to assess emotions in education	10.1007/978-3-319-29049-2_4	58
McPhetres (2019), COGN EMOT	Oh, the things you don't know: awe promotes awareness of knowledge gaps and science interest	10.1080/02699931.2019.1585331	54
Chevrier et al. (2019), LEARN INSTR	Exploring the antecedents and consequences of epistemic emotions	10.1016/j.learninstruc.2019.05.006	53
Anderson et al. (2020), J PERS	Are awe-prone people more curious? The relationship between dispositional awe, curiosity, and academic outcomes	10.1111/jopy.12524	49
Thacker et al. (2020), J EDUC PSYCHOL-a	Using persuasive refutation texts to prompt attitudinal and conceptual change.	10.1037/edu0000434	39
Muis et al. (2018b), CONTEMP EDUC PSYCHOL	Main and moderator effects of refutation on task value, epistemic emotions, and learning strategies during conceptual change	10.1016/j.cedpsych.2018.10.001	35

### 3.5 Geographical and institutional contribution

#### 3.5.1 Most productive countries

Top 10 countries in scientific production shown in Table 5 suggests that the United States leads with 72 publications, followed by Germany with 48, and China with 34. The United Kingdom and Australia follow closely with 30 and 29 publications respectively, while Canada contributes 27 publications. Other notable contributors include Italy (15), Finland (13), Switzerland (12), and Japan (9).

**Table 5:** Top 10 most productive countries based on the total number of publications.

Country	Total publication
USA	72
Germany	48
China	34
UK	30
Australia	29
Canada	27
Italy	15
Finland	13
Switzerland	12
Japan	9

**Figure 5:**
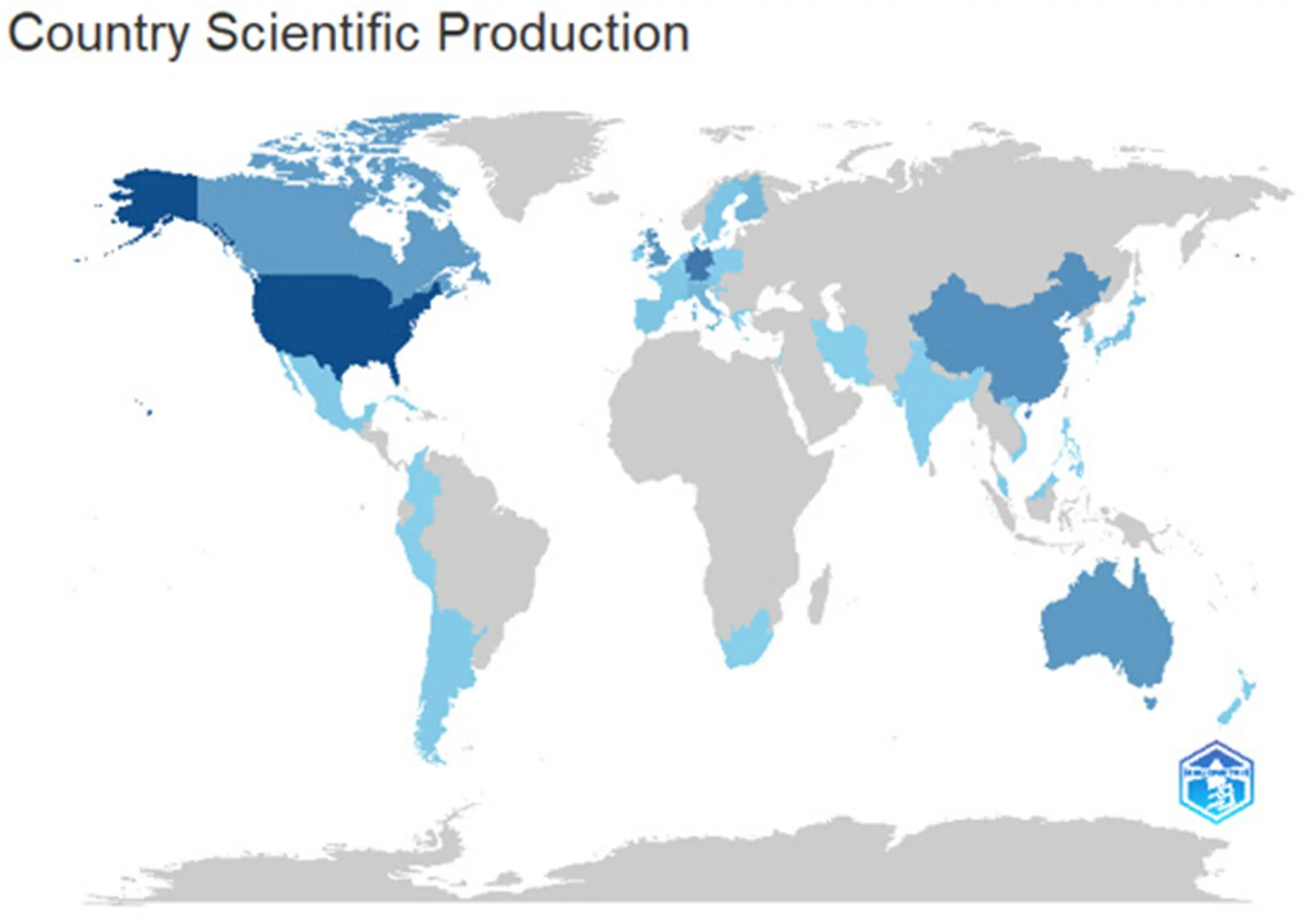
Map showing countries contributing to epistemic emotions research.

The geographical map, depicted in Figure 5 further emphasizes the United States as the global leader in research output, with darker shades indicating higher publication frequencies. Significant contributions are also observed from Germany, the United Kingdom, China, and Australia, highlighting the dominance of North America and Europe in epistemic emotions research, with emerging contributions from Asia, particularly China and Japan.

#### 3.5.2 Publication trend of top countries

**Figure 6:**
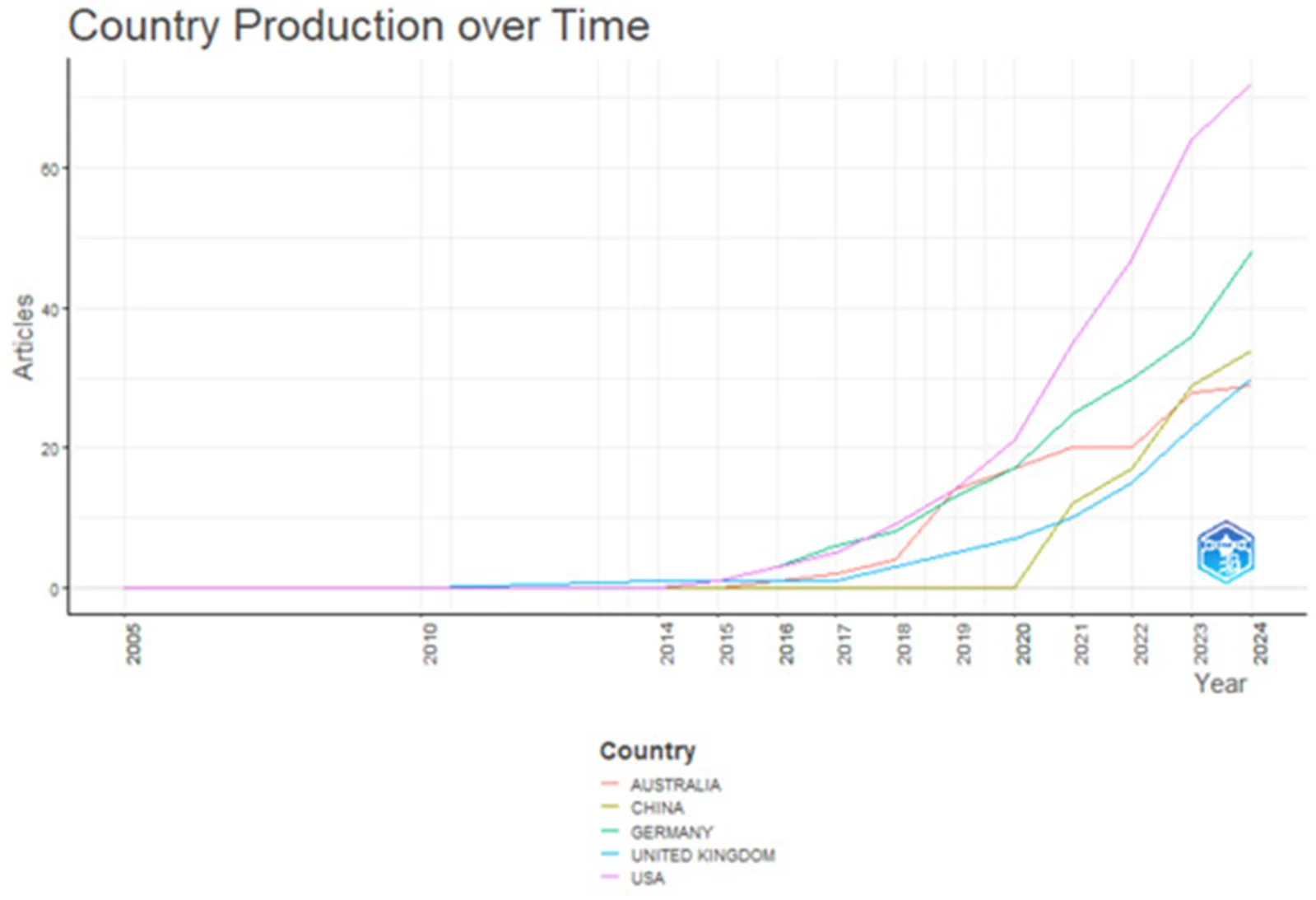
Publication trend of top five countries.

The publication trend of the top five countries, depicted in Figure 6 shows that research in epistemic emotions was limited before 2010. The United States initiated its research in 2015, experiencing rapid growth from 2018 onward and reaching 72 publications by 2024. Germany followed a steady upward trajectory beginning in 2016, achieving 48 publications by 2024. The United Kingdom showed early interest, starting in 2014 and progressing gradually until 2019, before increasing sharply to reach 30 publications by 2024. Australia entered the field in 2016, accelerating from 2019 to reach 29 publications by 2024. China joined later, with substantial contributions beginning in 2021 and rising quickly to 34 publications by 2024. This trend illustrates an expanding global interest and collaboration in epistemic emotions research, with leading contributions from North America, Europe, and the Asia-Pacific region.

#### 3.5.3 Most cited countries

**Figure 7:**
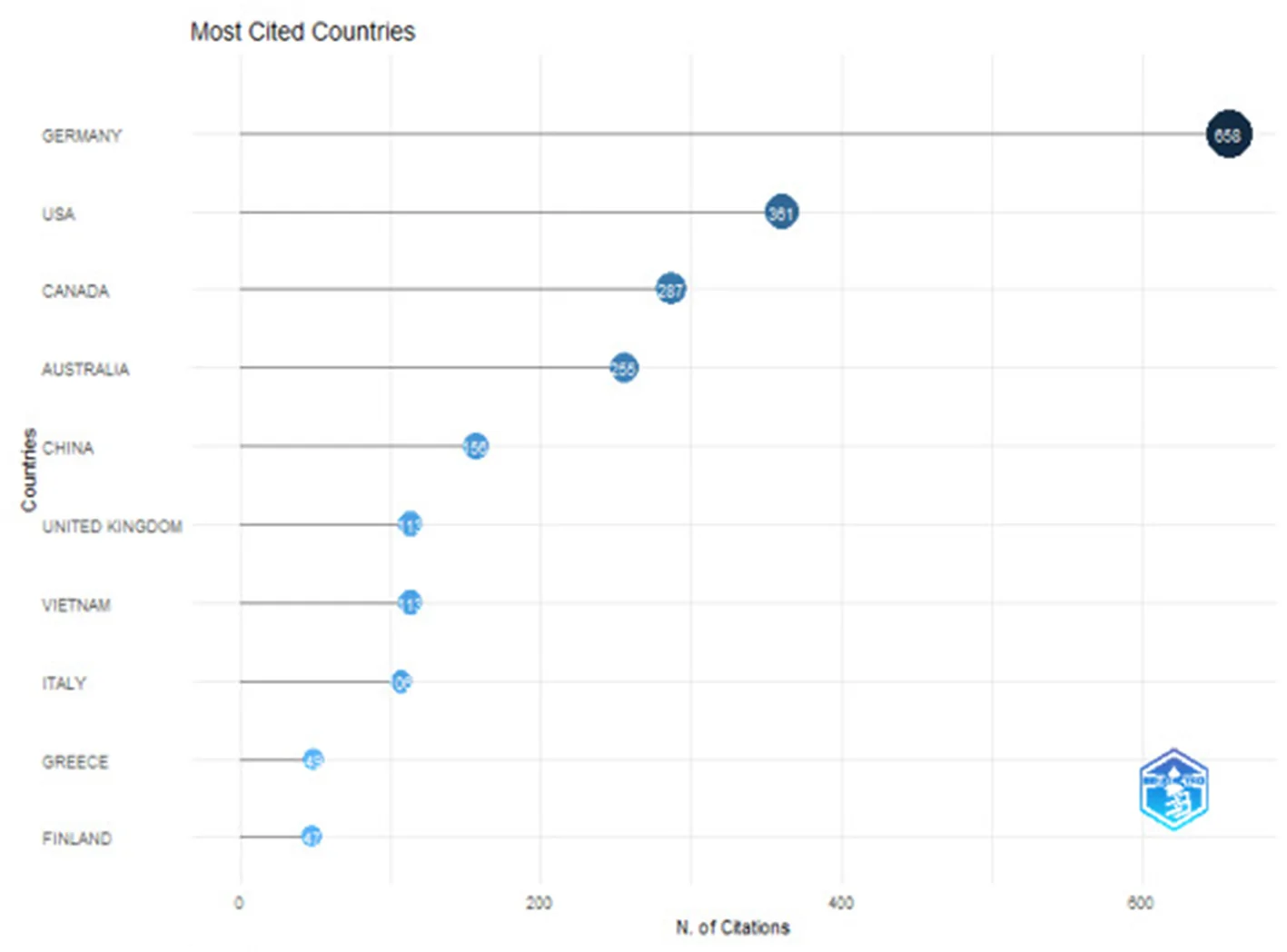
Most cited countries.

The 10 most cited countries, depicted in Figure 7, reveals that Germany leads with 658 total citations and the highest average article citations (36.6), indicating a significant impact in the field. The United States follows with 361 citations and an average of 11.3 citations per article, reflecting broad contributions. Canada and Australia also show strong influence with 287 and 255 citations, respectively, and high average citations per article, while China demonstrates growing prominence with 156 total citations. Other notable contributors include the United Kingdom, Vietnam, Italy, Greece, and Finland.

#### 3.5.4 Cross country collaboration

**Figure 8:**
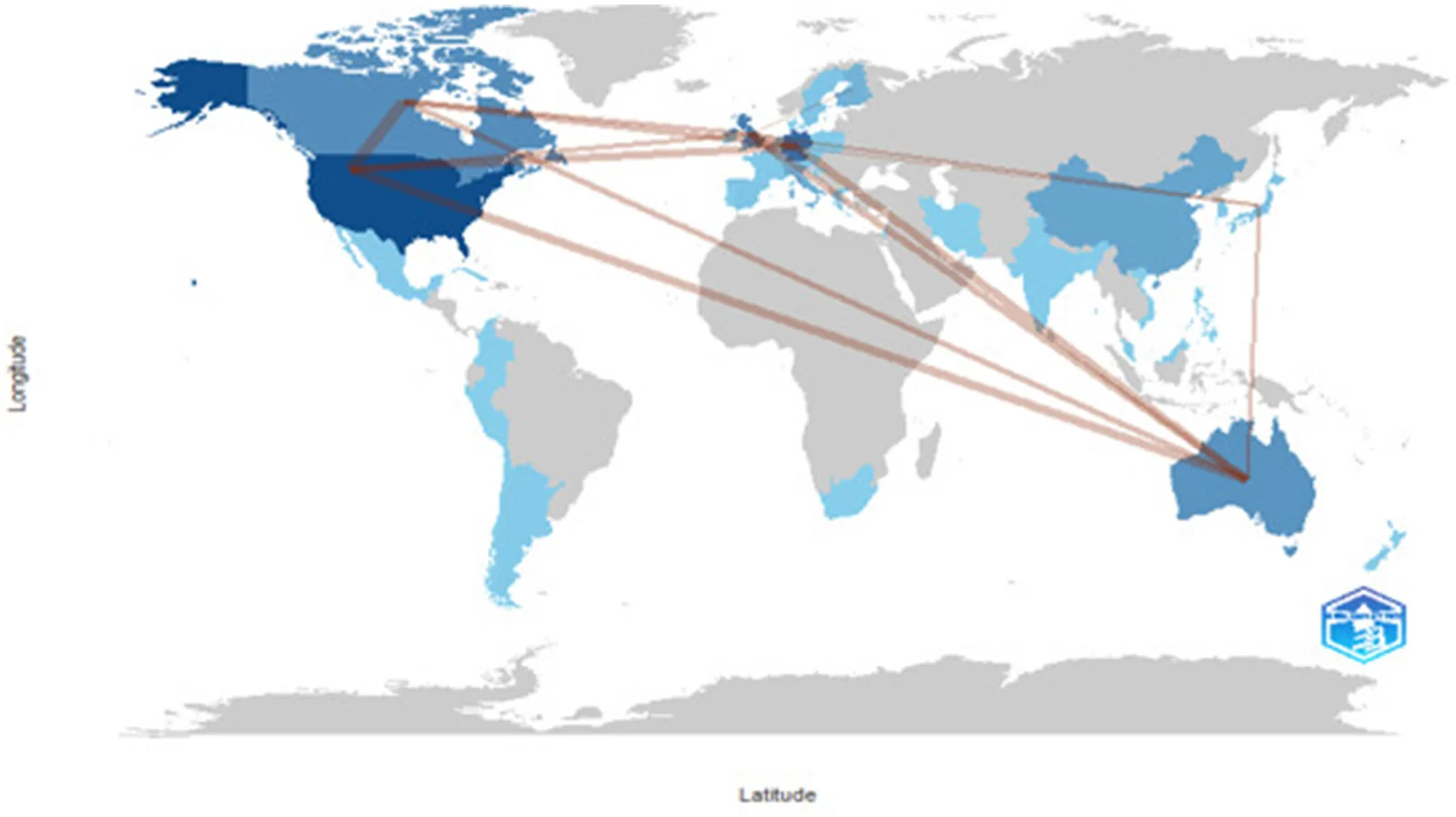
Cross-country collaboration network map.

The cross-country collaboration, shown in Table 6, indicates that the United States leads in both single-country publications (SCP) and multi-country publications (MCP), with 34.4% of its publications involving international collaboration. Germany follows with 22.2% MCP, indicating strong collaborative ties, especially with other European countries and the US. Australia has the highest percentage of MCPs (36.4%), reflecting a strong tendency for international collaboration, particularly with the US. Other notable countries with significant international partnerships include the United Kingdom, Canada, and China, each showing a blend of domestic and international research collaborations. The collaboration network figure (Figure 8) further illustrates these strong cross-regional ties, with dense connections between North America, Europe, and Australia, highlighting the global nature of epistemic emotions research.

**Table 6:** Cross-country collaboration in epistemic emotions research, showing each country's proportion of single-country (SCP) and multi-country (MCP) publications.

Country	Article	SCP	MCP	MCP%
USA	32	21	11	34.375
Germany	18	14	4	22.22
United Kingdom	14	11	3	21.42
China	12	11	1	8.33
Australia	11	7	4	36.36
Canada	10	5	5	50
Italy	10	7	3	30
Finland	8	6	2	25
Switzerland	4	3	1	25
Greece	3	3	0	0

#### 3.5.5 Most productive institutions

The Table 7 highlights the most active institutions in epistemic emotions research. It shows that McGill University leads with the highest number of publications (19 articles), followed closely by the University of Helsinki with 18 articles. University of Munich also stands out with 12 publications, indicating its significant contribution to the field. Australian Catholic University and the University of Southern California each have 10 articles, emphasizing their role in advancing research in this area. Other notable institutions include Tampere University (8 articles), The University of Edinburgh (seven articles), and Free University of Berlin, Ludwig-Maximilians-Universität München, and Monash University with six articles each. This distribution highlights the prominent global research contributions, especially from institutions in North America, Europe, and Australia, reflecting a widespread academic interest in epistemic emotions across various regions.

**Table 7:** Top 10 most productive institutions based on the number of publications.

Affiliation	Articles
MCGILL UNIVERSITY	19
UNIVERSITY OF HELSINKI	18
UNIVERSITY OF MUNICH	12
AUSTRALIAN CATHOLIC UNIVERSITY	10
UNIVERSITY OF SOUTHERN CALIFORNIA	10
TAMPERE UNIVERSITY	8
THE UNIVERSITY OF EDINBURGH	7
FREE UNIVERSITY OF BERLIN	6
LUDWIG-MAXIMILIANS-UNIVERSITÄT MÜNCHEN	6
MONASH UNIVERSITY	6

### 3.6 Keyword analysis and research themes

#### 3.6.1 Most frequent authors' keywords

The Top 10 Keywords, depicted in Table 8, highlight frequently occurring terms in epistemic emotions research. “Epistemic emotions” leads with 22 occurrences, highlighting the central focus of the field. Other prominent keywords, including “beliefs” (20), “students” (19), “achievement” (15), “knowledge” (14), “curiosity” (13), and “motivation” (13), suggest an emphasis on understanding how epistemic emotions interact with learners' cognitive beliefs, drive academic achievement, and influence knowledge acquisition. Keywords like “science” (12), and “affective states” (11) indicate an interest in exploring the role of epistemic emotions within scientific learning contexts, as well as their relationship with different affective states. Collectively, these keywords reveal core themes and emerging directions in the field, highlighting a focus on the interplay between emotions, cognition, and educational outcomes.

**Table 8:** Top 10 authors' keywords.

Words	Occurrences
Epistemic emotions	22
Beliefs	20
Students	19
Model	18
Achievement	15
Knowledge	14
Curiosity	13
Motivation	13
Science	12
Affective States	11

#### 3.6.2 Word cloud of most popular keywords plus

**Figure 9:**
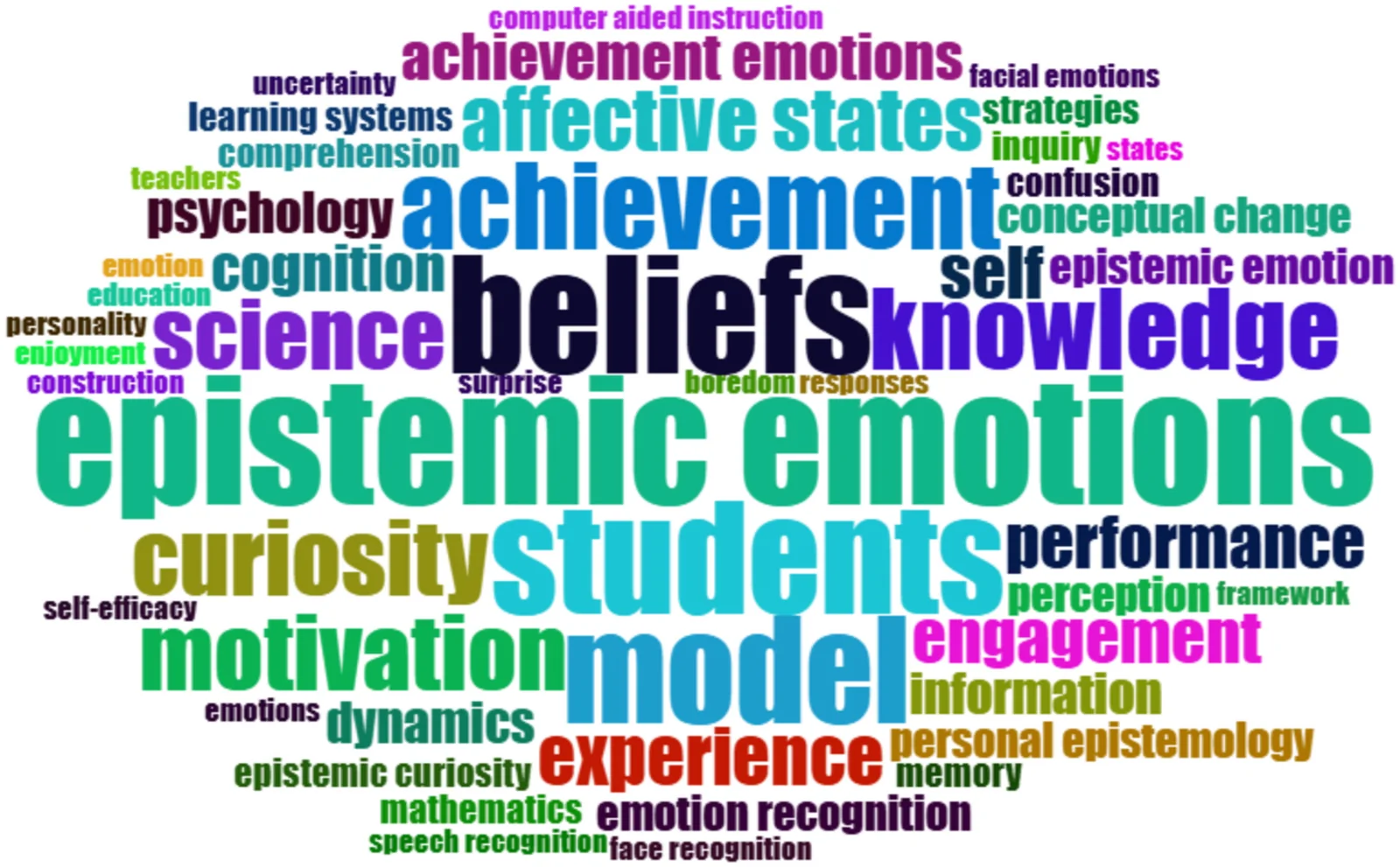
Word cloud of 100 most frequent keyword plus.

The word cloud, in Figure 9, visualizes the 100 most frequent keywords plus in epistemic emotions research, highlighting core themes and concepts in the field. Prominent terms like “epistemic emotions,” “beliefs,” “students,” “performance,” and “knowledge” reflect the focus on how epistemic emotions influence learning, belief formation, and academic performance. Keywords such as “curiosity,” “achievement,” “engagement,” and “motivation” emphasize the role of these emotions in driving exploration and interest in learning contexts. Overall, the word cloud illustrates the emphasis on understanding the psychological and emotional factors that impact students' engagement and achievement in learning environments.

#### 3.6.3 Keyword plus co-occurrence network

**Figure 10:**
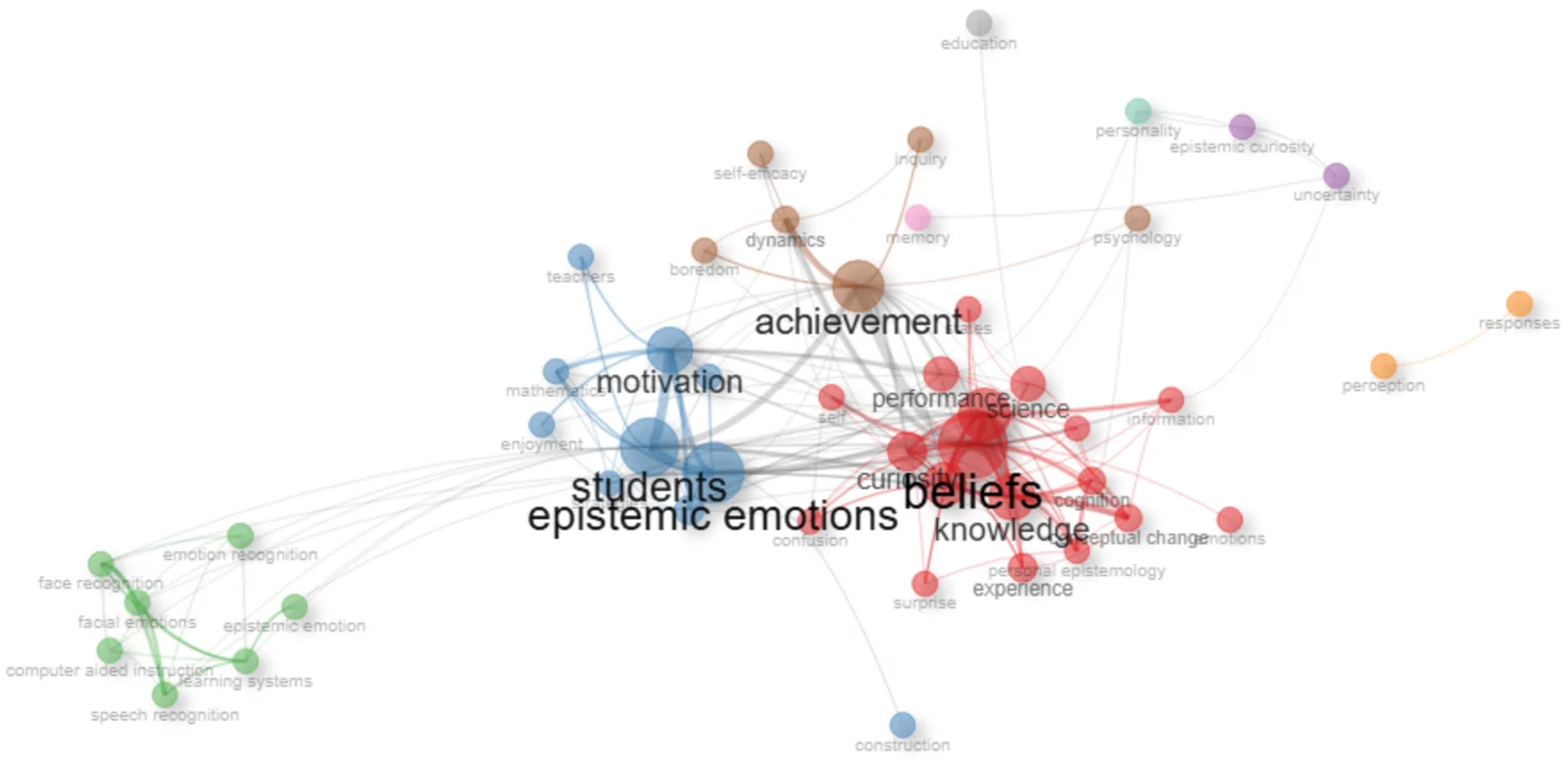
Co-occurrence network of keyword plus.

The co-occurrence network of popular keyword plus, depicted in Figure 10, illustrates the thematic structure within epistemic emotions research by mapping frequently co-occurring keywords. At the core, “beliefs,” “affective states,” and “epistemic emotions” form the central cluster, highlighting a primary focus on the role of emotional and belief-related states in cognitive processes and learning. This cluster connects with terms like “curiosity,” “confusion,” and “conceptual change,” suggesting a significant emphasis on how epistemic emotions drive motivation and learning adaptation. Surrounding clusters reveal additional themes, with achievement and motivation represented through keywords like “self-efficacy,” “motivation,” and “strategies,” indicating a strong link between epistemic emotions and academic performance. More specialized topics, such as “emotion recognition,” “facial emotions,” and “speech recognition” (in the green cluster), as well as isolated terms like “epistemic curiosity” and “uncertainty,” reflect niche or emerging areas of interest within the field. Overall, this network map provides a comprehensive view of the main research themes shaping epistemic emotions research.

### 3.7 Thematic mapping

**Figure 11:**
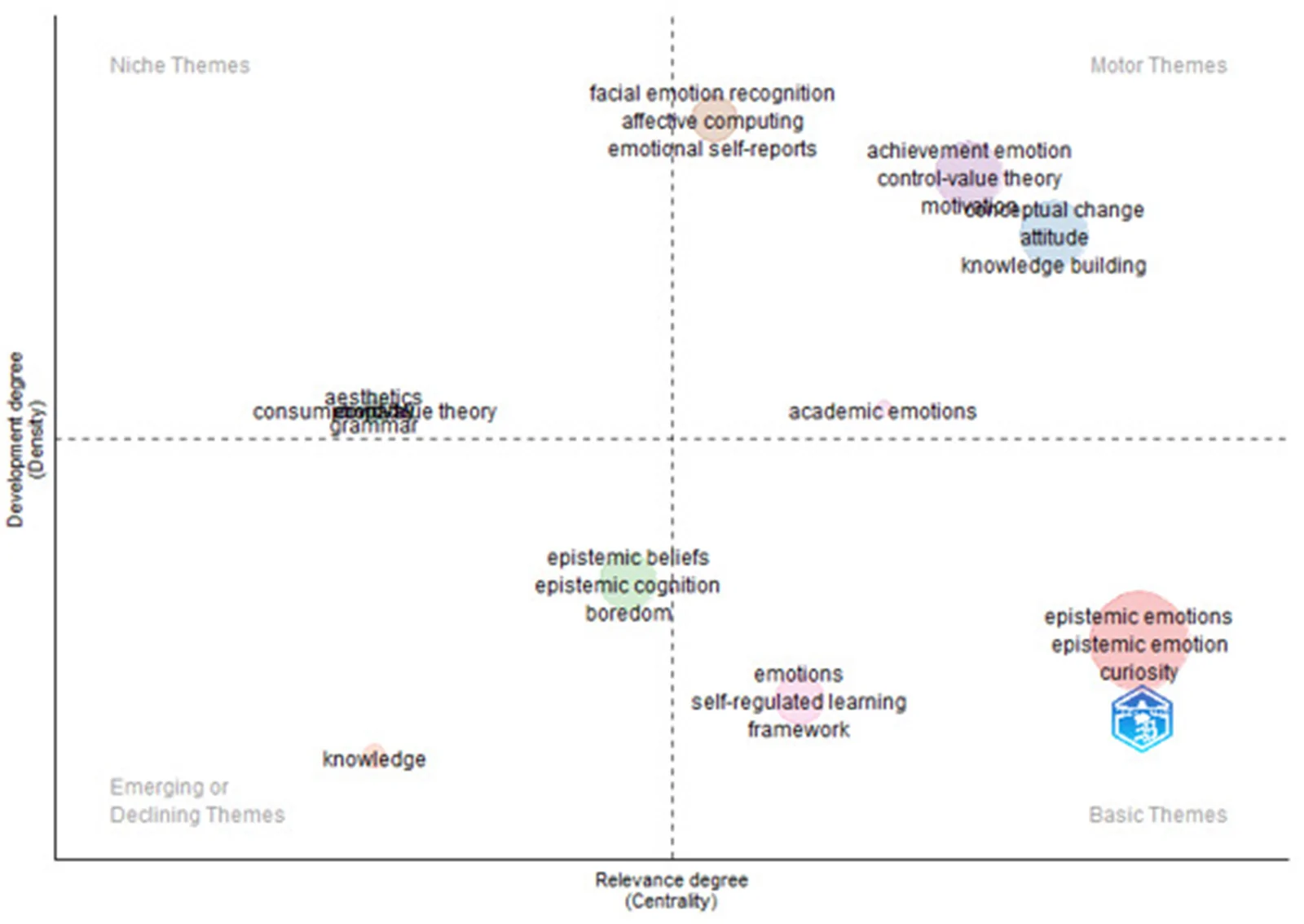
Thematic map of epistemic emotions research.

The thematic mapping in Figure 11 categorizes research themes in epistemic emotions into four quadrants, based on their relevance (centrality) and development (density). Motor themes include *academic emotions, achievement emotion, Control-Value Theory, motivation, conceptual change, knowledge building, facial emotion recognition, affective computing*, and *emotional self-reports*, indicating that these topics are well developed and central to the field. Basic themes comprise *epistemic emotions, curiosity*, and *emotions*, reflecting their foundational role in the literature. The emerging or declining themes include *epistemic beliefs, epistemic cognition, knowledge*, and *boredom*, whereas the niche theme quadrant represents specialized but less central areas of research. Overall, the thematic map provides an overview of the conceptual organization of epistemic emotions research.

## 4 Discussion

The findings of this bibliometric analysis offer a detailed overview of the evolution and current landscape of epistemic emotions research from its initial appearance in Scopus and Web of Science in 2005 through to 2024. This study highlights key trends and provides valuable insights, presenting a thorough understanding of the progress and focus areas within the field of epistemic emotions research.

The rapid growth of studies on epistemic emotions, particularly after 2017, reflects more than an increase in publication volume; it signifies the maturation of the field from a largely theoretical area of inquiry into an established empirical research domain. Earlier, the studies were primarily concerned with defining epistemic emotions and distinguishing them from other academic emotions. Therefore, they developed theoretical frameworks that explained how emotions arise during knowledge construction and cognitive conflict (Pekrun et al., 2006; Morton, 2010). Over the past decade, however, the research landscape has shifted toward investigating the functional role of epistemic emotions in learning, self-regulation, conceptual change, and academic achievement. This transition has been facilitated by the availability of validated measurement instruments, particularly the Epistemically-Related Emotion Scales (Pekrun et al., 2017a,b), which enabled researchers to move beyond conceptual discussions and systematically examine emotions such as curiosity, confusion, and surprise across diverse learning contexts. Therefore, the sharp increase in publications after 2017 appears to coincide with this methodological and theoretical consolidation, as well as with the growing recognition that effective learning involves the interaction of cognitive, motivational, and affective processes rather than cognition alone (Pekrun, 2022; Muis et al., 2018a,b; Pekrun and Stephens, 2012). Moreover, the predominance of empirical studies in the present dataset (81.6%) indicates that the field has progressed beyond theory building toward evidence-based investigations across classrooms, digital learning environments, and technology-enhanced educational settings (Muis et al., 2018a,b; Vogl et al., 2019). Collectively, these findings suggest that epistemic emotions have evolved from a niche topic within educational psychology into a central construct for understanding how learners engage with uncertainty, regulate knowledge acquisition, and construct meaningful learning experiences.

The authorship and collaboration patterns indicate that the intellectual development of epistemic emotions research has been shaped by a relatively concentrated group of influential scholars, particularly Reinhard Pekrun, Krista Muis, and Gale Sinatra. They have played pivotal role in establishing the conceptual foundations of the field, advancing theoretical frameworks such as Control-Value Theory and, developing validated measurement instruments, and demonstrating the role of epistemic emotions in learning, conceptual change, and self-regulated learning. The prominence of these scholars indicates that the field has achieved a considerable degree of theoretical coherence, with subsequent research building on common conceptual models rather than opposing theoretical models. At the same time, the observed international co-authorship rate (25.41%) and the presence of distinct collaboration clusters indicate that epistemic emotions research has evolved through strong multi-institutional partnerships, facilitating methodological rigor and the exchange of interdisciplinary expertise. However, the collaboration network still shows highly dispersed research groups, indicating that knowledge generation is still focused on a small number of well-established research communities. Increased collaboration among these clusters, such as involving researchers from underrepresented geographical regions and complementary disciplines such as neuroscience, learning analytics, and educational technology, could promote theoretical diversification and expand the application of epistemic emotions research across diverse educational and cultural contexts.

The distribution of publications across leading journals provides important insights into the intellectual positioning of epistemic emotions research. The prominence of journals such as *Frontiers in Education, Frontiers in Psychology, Journal of Educational Psychology*, and *Learning and Instruction* suggests that the field continues to be anchored primarily within educational psychology, where understanding the interaction between cognition, motivation, and emotion has become a major research area. At the same time, the presence of influential journals such as *Cognition and Emotion, Computers in Human Behavior*, and *Contemporary Educational Psychology* demonstrates the gradual expansion of epistemic emotions research into allied disciplines, including cognitive psychology, educational technology, and human-computer interaction. This publication pattern reflects the increasingly interdisciplinary nature of the field, where epistemic emotions are also viewed as mechanisms relevant to technology enabled learning, digital environments, and adaptive educational systems. Furthermore, the moderate average citation impact of the publications suggests that the literature has established a stable and influential knowledge base upon which subsequent empirical and theoretical studies continue to build. The relatively limited representation of journals from neuroscience, learning sciences, and artificial intelligence indicates that opportunities remain for greater interdisciplinary integration. Expanding collaborations across these disciplines could advance the development of multimodal approaches for understanding, measuring, and supporting epistemic emotions in increasingly complex learning environments. As learning increasingly shifts toward digital, adaptive, and AI-supported environments, future research is likely to benefit from stronger engagement with fields such as learning analytics, affective computing, cognitive neuroscience, and artificial intelligence. Such interdisciplinary integration may facilitate the development of more comprehensive theoretical models and multimodal methods for investigating epistemic emotions across diverse educational settings.

The geographical distribution of publications indicates that epistemic emotions research has been concentrated primarily in North America, Europe, and, more recently, Asia, with the United States, Germany, and China emerging as the leading contributors. This pattern likely reflects the strong research works in educational psychology, cognitive science, and learning sciences within these regions, as well as sustained institutional investment in educational research. The prominence of institutions such as McGill University, the University of Helsinki, and the University of Munich further demonstrates that the field has developed around internationally recognized research centers that have consistently contributed to theoretical advancement, methodological innovation, and empirical validation. While this concentration has facilitated the development of a coherent and cumulative body of knowledge, it also highlights an important geographical imbalance. The relatively limited contribution from developing regions, including Africa, Latin America, and South Asia, suggests that current evidence is derived predominantly from Western educational systems and sociocultural contexts. Given that epistemic emotions are shaped by learners' beliefs, educational practices, and cultural norms, greater geographical diversity is essential for examining the cross-cultural validity of existing theoretical frameworks and expanding their applicability across different educational settings. Future research should therefore encourage broader international collaboration and greater participation from underrepresented regions to develop a more globally representative understanding of epistemic emotions.

The thematic map provides important insights into the conceptual structure of epistemic emotions research by illustrating the relative importance and developmental status of the field's major research themes. The prominence of achievement emotions, Control-Value Theory, motivation, conceptual change, and knowledge building as motor themes indicates that these topics are both well developed and central to the intellectual structure of the field. Rather than focusing solely on defining epistemic emotions, contemporary research increasingly examines how these emotions influence learning processes, motivation, and academic achievement. In particular, the prominence of Control-Value Theory highlights its role as a dominant framework for explaining how learners' appraisals of control and value shape epistemic emotions and subsequently influence cognitive engagement and learning outcomes (Pekrun et al., 2017a,b). Likewise, the positioning of conceptual change and knowledge building as motor themes reflects the growing integration of epistemic emotions within contemporary learning theories that emphasize active knowledge construction, cognitive conflict, and meaningful learning (Muis et al., 2018a,b). Collectively, these findings suggest that epistemic emotions have become firmly embedded within broader theoretical models of learning rather than being investigated as isolated emotional experience.

Interestingly, methodological themes such as facial emotion recognition, affective computing, and emotional self-reports also appear within the motor-theme quadrant, suggesting that methodological innovation is becoming an important driving force in epistemic emotions research. This pattern reflects the increasing integration of educational psychology with artificial intelligence, learning analytics, cognitive neuroscience, and human-computer interaction. Rather than relying exclusively on traditional self-report measures, researchers are increasingly adopting multimodal approaches to detect and understand epistemic emotions through facial expressions, physiological signals, and computational techniques (Arguel et al., 2019; Dubey and Griffiths, 2020). These developments suggest a broader shift from viewing epistemic emotions primarily as psychological constructs to investigating them as measurable processes that can inform technology-enhanced learning environments and adaptive educational systems (Murayama et al., 2019).

At the same time, the thematic map highlights epistemic beliefs, epistemic cognition, boredom, and knowledge, within the emerging or declining themes quadrant. Rather than considering their position as evidence of declining importance, these themes may represent areas requiring stronger theoretical integration within the broader epistemic emotions literature. For example, constructs like epistemic beliefs and epistemic cognition have often been studied separately from epistemic emotions. Their position in this quadrant suggests opportunities for future research to develop more integrated theoretical models that explain how learners' beliefs about knowledge, cognitive processes, and emotional experiences interact during learning. Similarly, the placement of boredom indicates that its relationship with epistemic emotions and learning processes remains comparatively underexplored and warrants further investigation.

Overall, the thematic configuration suggests that epistemic emotions research is characterized by a strong theoretical foundation centered on learning, motivation, and knowledge construction while simultaneously expanding toward technologically enabled methods and interdisciplinary applications. This thematic map highlights the continued evolution of epistemic emotions research toward a more integrated, interdisciplinary, and application-oriented research domain.

## 5 Conclusion and future directions

This bibliometric review provides a comprehensive synthesis of epistemic emotions research published between 2005 and 2024. The findings demonstrate that the field has developed into an increasingly interdisciplinary research domain, characterized by sustained growth in scholarly output, expanding collaboration networks, and growing integration across educational psychology, cognitive science, neuroscience, and technology-enhanced learning. By mapping the intellectual structure, influential contributors, and thematic organization of the literature, this review offers a consolidated understanding of the field and identifies emerging directions for future research.

**Figure 12:**
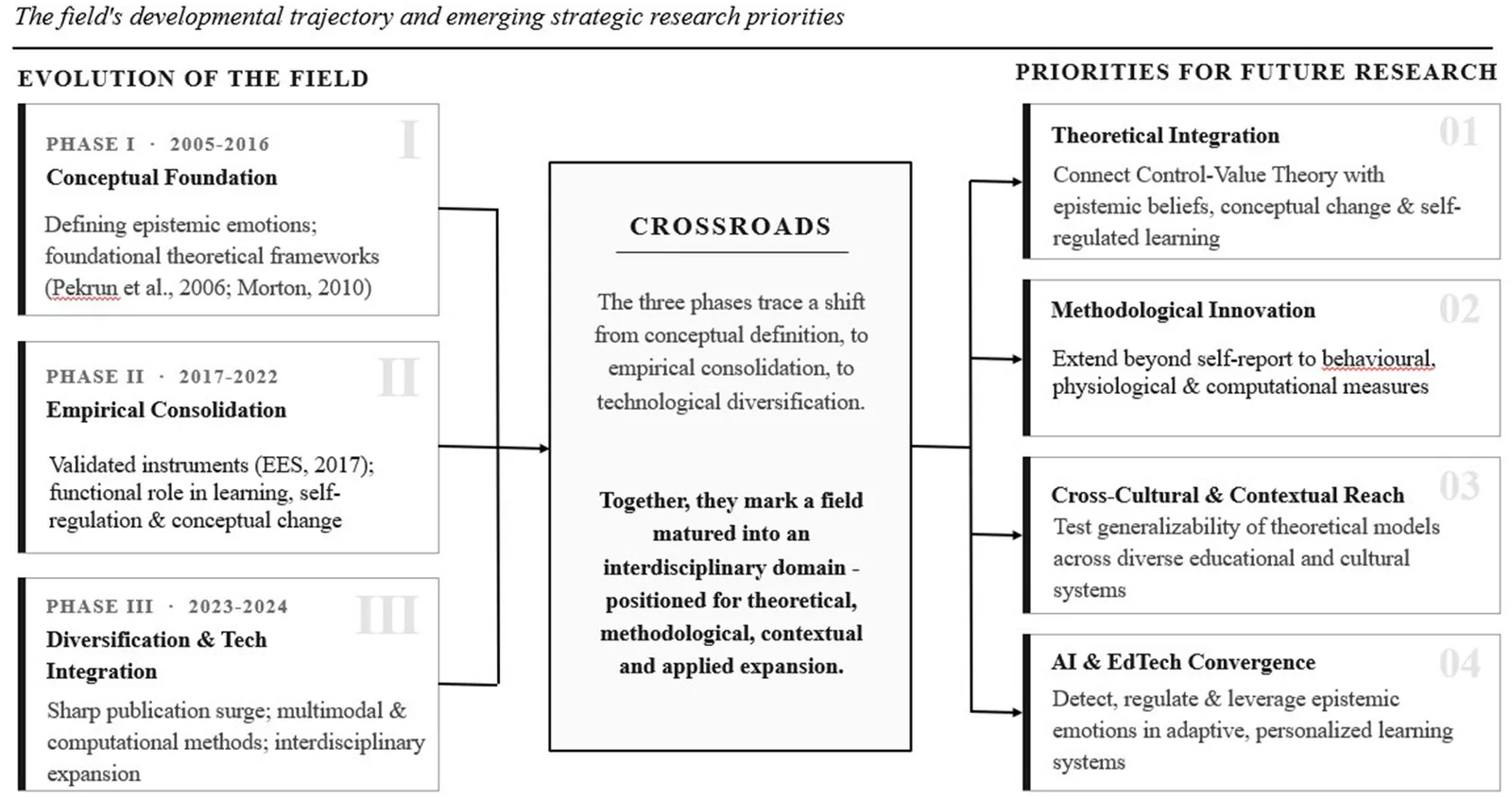
A conceptual roadmap of epistemic emotions research (2005–2024).

Figure 12 presents a conceptual roadmap that visually integrates the evolution of epistemic emotions research with its emerging research directions. By synthesizing the field's developmental trajectory and four strategic research priorities, the roadmap provides an integrative framework for the proposed future research agenda. Building on this conceptual synthesis, four strategic research priorities emerge to guide the next phase of epistemic emotions research.

First, greater theoretical integration is needed to connect dominant frameworks, such as Control-Value Theory, with related constructs such as, epistemic beliefs, conceptual change, and self-regulated learning to develop a more comprehensive understanding of how epistemic emotions influence learning and knowledge construction. Second, methodological innovation should extend beyond traditional self-report measures by incorporating behavioral, physiological, and computational approaches, particularly as affective computing, emotion recognition, and learning analytics continue to advance. Third, broader cross-cultural and contextual investigations are needed to examine the generalizability of existing theoretical models across diverse educational systems and learning environments. Finally, the growing convergence of epistemic emotions with educational technology and artificial intelligence presents significant opportunities to investigate how these emotions can be detected, regulated, and leveraged within adaptive and personalized learning systems. Together, these strategic priorities provide a research agenda for the future of epistemic emotions research, promoting stronger theoretical integration, methodological innovation, interdisciplinary collaboration, and broader educational application.

## Data Availability

The original contributions presented in the study are included in the article/supplementary material, further inquiries can be directed to the corresponding author.
